# A rapid increase in lysophospholipids after geranylgeranoic acid treatment in human hepatoma-derived HuH-7 cells revealed by metabolomics analysis

**DOI:** 10.1016/j.bbrep.2021.101176

**Published:** 2021-11-24

**Authors:** Yoshihiro Shidoji, Chieko Iwao

**Affiliations:** Molecular and Cellular Biology, Graduate School of Human Health Science, University of Nagasaki, Nagayo, Nagasaki, 851-2195, Japan

**Keywords:** Geranylgeranoic acid, Lysophospholipids, Hepatoma, Metabolomics, Cell death, ATRA, all-*trans* retinoic acid, D-MEM, Dulbecco’s modified Eagle’s medium, ENPP2, ectonucleotide pyrophosphatase/phosphodiesterase 2, FBS, fetal bovine serum, GGA, geranylgeranoic acid, GSDMD, gasdermin D, HMDB, Human Metabolome Database, KEGG, Kyoto Encyclopedia of Genes and Genomes, LCAT, lecithin cholesterol acyltransferase, LIPC, lipase C, LPA, lysophosphatidic acid, LPC, lysophosphatidylcholine, LPCAT, LPC acyltransferase, LPE, lysophosphatidylethanolamine, LPL, lysophospholipid, OPLS-DA, orthogonal partial least squares-discriminant analysis, PCA, principal component analysis, PLA2, phospholipase A2, Q-Tof/MS, quadrupole time-of-flight type mass spectrometry, SPH, second primary hepatoma, TLR4, toll-like receptor-4, UPLC, ultra-performance liquid chromatography, UPR^ER^, unfolded protein response or endoplasmic reticulum stress response, VIP, variable importance in the projection

## Abstract

Geranylgeranoic acid (GGA) was developed as a preventative agent against second primary hepatoma, and was reported to induce cell death in human hepatoma cells via Toll-like receptor 4 (TLR4)-mediated pyroptosis. We recently reported that GGA is enzymatically biosynthesized from mevalonic acid in human hepatoma-derived HuH-7 cells and that endogenous GGA is found in most rat organs including the liver. An unbiased metabolomics analysis of ice-cold 50% acetonitrile extracts from control and GGA-treated cells was performed in this study to characterize the intracellular metabolic changes in GGA-induced pyroptosis and to analyze their relationship with the mechanism of GGA-induced cell death. The total positive ion chromatograms of the cellular extracts in ultra-performance liquid chromatography coupled with quadrupole time-of-flight mass spectrometry were apparently unchanged after GGA treatment, but an orthogonal partial least squares-discriminant analysis score plot clearly discriminated the intracellular metabolite profiles of GGA-treated cells from that of control cells. S-plot analysis revealed 15 potential biomarkers up-regulated by 24-h GGA treatment according to their variable importance in the projection value of more than 1, and the subsequent metabolomics analysis identified nine of these metabolites as a group of lysophospholipids containing lysophosphatidylcholine with C16:0, C20:4, or C20:3 fatty acids. The possible roles of these lysophospholipids in GGA-induced pyroptosis are discussed.

## Highlights

• Metabolomics analysis was performed on geranylgeranoic acid (GGA)-treated cells.
• Total positive ion chromatograms were apparently similar after GGA treatment.
• The OPLS-DA score plot distinguished the GGA-treated cells from control cells.
• The S-plot analysis revealed GGA-induced upregulation of lysophospholipids.
• The possible roles of lysophospholipids in GGA-induced pyroptosis are discussed.

## 1 Introduction

In 1995, we discovered a group of "acyclic retinoids" without a cyclic structure, which have ligand activity on retinoid receptors [1]. At the beginning of our research, we found that acyclic retinoids do indeed act as ligands for nuclear retinoid receptors and have differentiation-inducing effects such as enhanced albumin production in some human hepatoma cell lines [1,2]. Furthermore, acyclic retinoid significantly induced neural differentiation of the human neuroblastoma cell line SH-SY5Y, as did the natural active retinoid all-*trans*-retinoic acid (ATRA) [3].

Geranylgeranoic acid (GGA or all-*trans*-3,7,11,15-tetramethyl-2,6,10,14-hexadecatetraenoic acid) and its 4,5-didehydro derivative (peretinoin or all-*trans*-3,7,11,15-tetramethyl-2,4,6,10,14-hexadecapentaenoic acid) are the most well-studied acyclic retinoids so far as a preventive agent for second primary hepatoma (SPH) [4,5]. Indeed, peretinoin was shown to have the potential to prevent SPH in randomized, placebo-controlled phase II/III clinical trials [6,7]. Using human hepatoma-derived cell lines, we found that both GGA and peretinoin induced tumor-specific cell death, which may be involved in the suppression of SPH [8,9]. We have reported that GGA induces cell death in human hepatoma cells via the unfolded protein response (UPR^ER^) and an incomplete response of autophagy [10,11]. Most recently, we concluded that GGA induces Toll-like receptor 4 (TLR4)-signaled pyroptosis in human hepatoma cells [12].

GGA causes activation of caspase-4 via TLR4-mediated UPR^ER^, resulting in cleavage of gasdermin D (GSDMD) and the translocation of its N-terminal fragment to the plasma membrane leading to the formation of the GSDMD pore [12]. Another TLR4 signaling causes increased reactive oxygen species production in the mitochondria, leading to the transfer of NF-κB to the nucleus and priming the NLRP3 inflammasome. Then, the influx of extracellular Ca^2+^ causes inflammasome activation, which in turn activates caspase-1, leading to extensive cleavage of GSDMD and completing pyroptosis [12]. Given that autophagy can inactivate the inflammasome [13], we believe that GGA-induced incomplete response of autophagy [10] may support the activation of caspase-1 by avoiding the inactivation of the inflammasome.

Although we have gained some understanding of the signal transduction pathways in GGA-induced cell death of human hepatoma cells, we still have only limited knowledge of the changes in intracellular metabolites of GGA-treated cells. We previously reported that during GGA-induced cell death, GGA shifts the energy state of human hepatoma-derived HuH-7 cells from aerobic glycolysis to mitochondrial respiration via up-regulation of ‘TP53-induced glycolysis regulatory phosphatase’ (TIGAR) and ‘synthesis of cytochrome C oxidase 2’ (SCO2) proteins [14]. In fact, we confirmed that GGA induced a time-dependent increase in the cellular contents of fructose 6-phosphate and decreased fructose 1,6-diphosphate. Furthermore, a preliminary metabolomics analysis revealed that GGA rapidly induced spermine accumulation [14].

In the present study, we decided to further perform the metabolomics analysis of HuH-7 cells to search for metabolites that respond rapidly and reliably to GGA. As a result, an orthogonal partial least squares-discriminant analysis (OPLS-DA) score plot distinguished GGA-treated cells from control cells, and S-plot analysis revealed that GGA induced the up-regulation of several lysophospholipid (LPL) species. The possible roles of the up-regulated LPLs in GGA-induced pyroptosis are discussed.

## 2 Materials and methods

### 2.1 Cell culture

HuH-7 cells were obtained from RIKEN BioResource Center (Tsukuba, Japan), and cultured in high-glucose Dulbecco’s modified Eagle’s medium (D-MEM; Wako, Osaka, Japan) containing 5% fetal bovine serum (FBS; Hyclone, Thermo Fisher Scientific, Waltham, MA).

### 2.2 GGA treatment

The cells were inoculated in 9-cm dishes, at a density of 2.5 × 10^6^ cells/dish and cultured with D-MEM containing 5% FBS for 2 days. Thereafter, the medium was replaced with FBS-free D-MEM 1 day before GGA addition to efficiently induce cell death in 24 h [8]. Since the LD_50_ of GGA for HuH-7 cells is in the range of 5–10 μM [10,11], GGA (10 mM in ethanol) was added at a final concentration of 10 μM for 2–24 h. Ethanol was added as a control at 0.1% (v/v).

### 2.3 Extraction of metabolites

The procedure for the preparation of cell extracts has already been described in detail in our paper [14]. Here, it is briefly presented below. After GGA treatment, the cells were quenched by replacement of the conditioned medium with ice-cold 0.9% (w/v) NaCl. Cell pellets were extracted in ice-cold 50% (v/v) aqueous acetonitrile (LC-MS Chromatosolv®; Fluka, Sigma-Aldrich, St. Louis, MO, USA), lyophilized, dissolved in water and stored frozen at −80°C until analysis.

Thawed and filtered samples were analyzed by ultra-performance liquid chromatography (UPLC) and quadrupole time-of-flight type mass spectrometric apparatus (Xevo™ Q-Tof/MS, Nihon Waters).

### 2.4 UPLC

Reversed-phase chromatographic separation was performed on a 2.1 × 100 mm ACQUITY™ UPLC® BEH C18, 1.7 μm column at 40°C with the mobile phase consisted of water containing 0.1% formic acid (HPLC grade, Wako) as solvent A and acetonitrile containing 0.1% formic acid as solvent B. Up to 0.5 min after sample injection, the volume proportion of acetonitrile in eluent was kept at 1% and linearly increased to 95% in 10 min at a constant flow rate of 0.3 mL/min. Then, acetonitrile was kept at 95% for 4 min. A 10-μL aliquot of each sample was injected automatically onto the column three times.

### 2.5 Q-Tof/MS

In-line mass spectrometry was performed on a Waters Xevo™ ESI-Q-Tof/MS apparatus, which was calibrated with sodium formate and lock-sprayed with Leu-enkephalin (Nihon Waters) each time, operating in positive ion mode. The operating conditions for Q-Tof/MS have already been described in detail [14]. 1-Palmitoyl-*sn*-glycero-3-phosphocholine (synthetic, ≥99%, Sigma-Aldrich) was used as the standard for lysophosphatidylcholine (LPC(16:0)).

### 2.6 Data collection, processing, and multivariate statistical analysis

Raw chromatogram data of UPLC/Q-Tof/MS were initially converted to NetCDF (network common data file) formatted by Databridge using MarkerLynx® software (Waters). The peak width at 50% height was set to 3 s. Other parameters were all in default settings. Detected and matched peaks with retention time and *m/z* and their corresponding intensities were electronically exported to an Excel table.

Principal component analysis (PCA) and OPLS-DA models were constructed to reproduce the differences in metabolites between GGA-treated and ethanol-treated control cells, using MarkerLynx® XS software. Potential biomarkers for GGA-treated cells were selected according to their variable importance in the projection (VIP) value of more than 1 (>1) in the S-plot.

## 3 Results

### 3.1 Metabolomic changes in GGA-treated cells

**Fig. 1:**
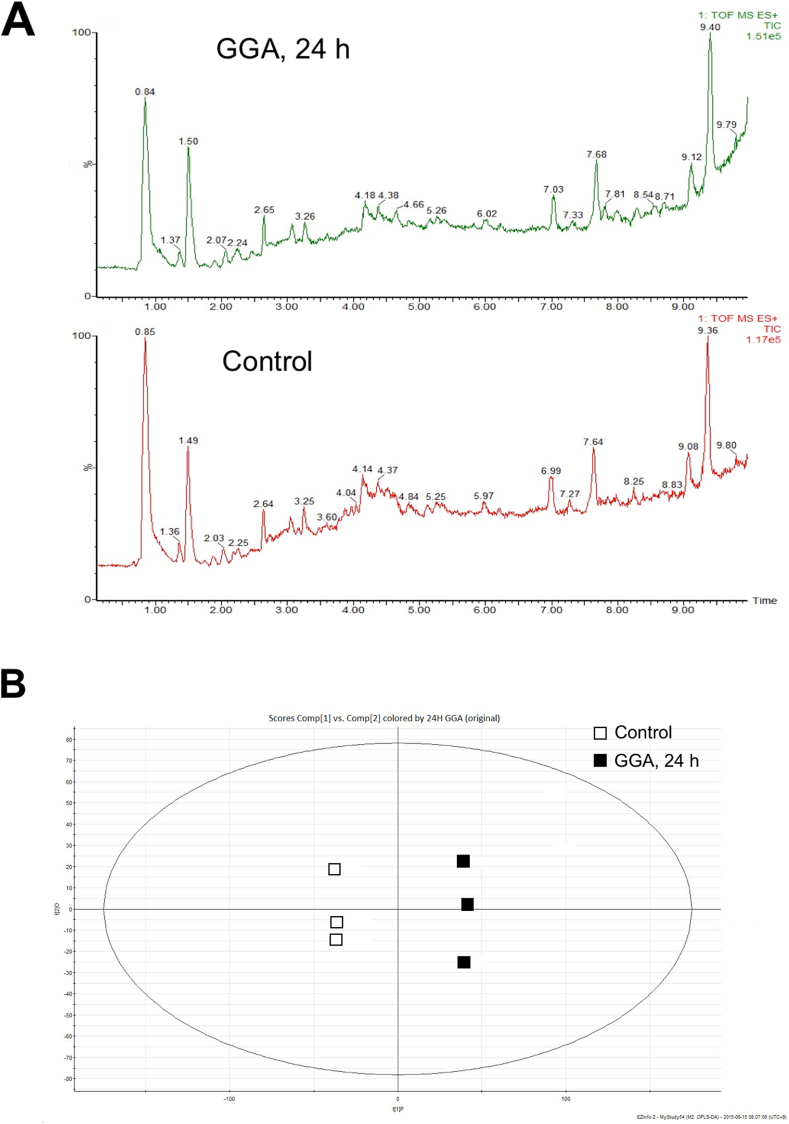
**A**: Representative UPLC/Q-Tof/MS total positive ion chromatograms of 24-h GGA-treated HuH-7 cell extracts (upper panel) and 0-h control cell extracts (lower). **B**: Score plot in OPLS-DA multivariate analysis of the data of UPLC/Q-Tof/MS. X-axis shows the variation between groups and Y-axis shows the variation within groups. Open squares show 0-h control and closed squares show 24-h GGA-treated cells in triplicate.

Although total positive ion (*m/z* 80 to 1000) chromatograms were apparently similar between GGA-treated cell and control cell extracts (Fig. 1A), the OPLS-DA score plot clearly distinguished the GGA-treated cells from the control cells in triplicate (Fig. 1B), suggesting that 10 μM GGA alters the profile of intracellular metabolites after 24-h treatment.

**Fig. 2:**
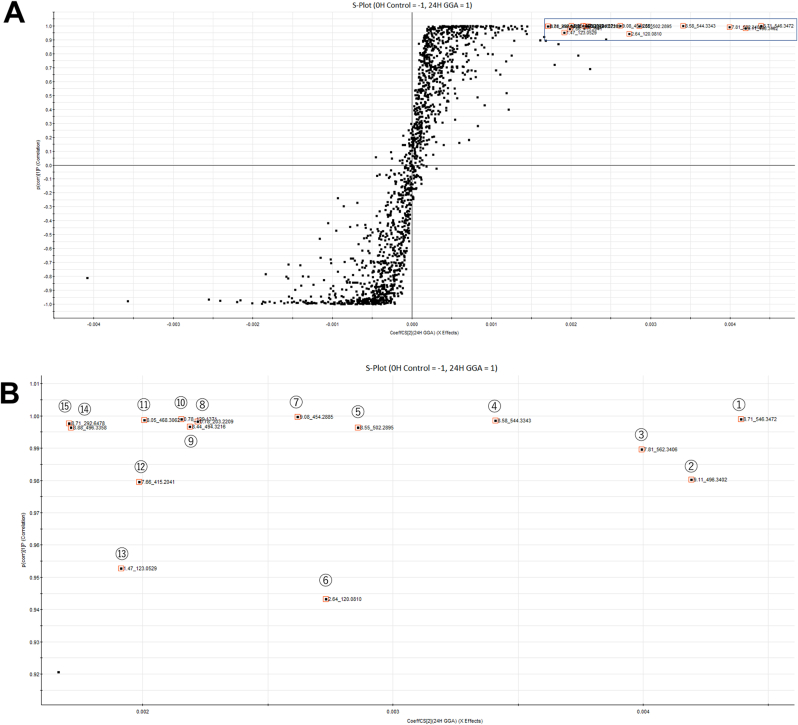
**A**: S-plot analysis. Potential biomarkers for GGA-treated cells were selected according to their VIP value of more than 1 and are marked by red squares. X-axis represents the magnitude of each variable and Y-axis represents the reliability of each variable. Twenty-four-hour GGA-treated HuH-7 cell extracts vs control cell extracts. **B**: The area enclosed by the black line square in the upper-right corner of panel A is shown enlarged. Dots surrounded by red squares are numbered in order from the most to the least variable, and are indicated by circled numbers. (For interpretation of the references to colour in this figure legend, the reader is referred to the Web version of this article.)

Then the OPLS-DA loadings S-plot analysis clearly visualized the biomarker candidates for GGA effects in the upper right of the first quadrant (Fig. 2A). In particular, the spots indicated by red squares indicated that the VIP values were greater than 1, which were selected as potent positive biomarkers for GGA-treated cells. All these selected spots with measured accurate masses were dropped into a database survey connected to Internet databases (KEGG: Kyoto Encyclopedia of Genes and Genomes <http://www.genome.jp/kegg/>, HMDB: Human Metabolome Database <http://www.hmdb.ca/>, and ChemSpider <http://www.chemspider.com/>) to identify each metabolite, and thus far, we have been able to identify most of the spots surrounded by the red squares only in the first quadrant.

### 3.2 Up-regulation of LPLs by GGA treatment

The portion detected in the first quadrant of the S plot (Fig. 2A) is magnified in Fig. 2B, in which each spot is numbered in the order of magnitude of its contribution to the variation between the control and GGA-treated cells. Of the 15 biomarkers (Figs. 2B), 11 were identified as metabolites by matching their protonated accurate masses detected in the three databases described above. Surprisingly and interestingly, nine of the 11 identified metabolites were all LPLs, and the remaining two were spermine and nicotinamide (Table 1). Of the LPL molecular species detected, seven were LPC and the remaining two were lysophosphatidylethanolamine (LPE).

**Table 1:** Highly and reliably up-regulated biomarkers in 24-h GGA-treated cells.

Spot no.	Time (min)	Accurate mass measured		Molecular formula assigned	Common name (with fatty acid)	Exact mass^a^ calculated	AM-EM^b^ calculated		HMDB^c^ ID
①	8.71		546.3472	C28H52NO7P	LPC(20:3)	545.34814	0.99906	HMDB0010394	
②	9.11		496.3402	C24H50NO7P	LPC(16:0)	495.33249	1.00771	HMDB0240262	
③	7.81		562.3406	C28H52NO8P	LPC(OH20:3)	561.34306	0.99754	–	
④	8.58		544.3343	C28H50NO7P	LPC(20:4)	543.33249	1.00181	HMDB0010395	
⑤	8.55		502.2895	C25H44NO7P	LPE(20:4)	501.28554	1.00396	HMDB0011487	
⑥	2.64		120.0810	?	–	–	–	–	
⑦	9.08		454.2885	C21H44NO7P	LPE(16:0)	453.28554	1.00296	HMDB0011503	
⑧	0.78		203.2209	C10H26N4	Spermine	202.21575	1.00515	HMDB0001256	
⑨	8.44		494.3216	C24H48NO7P	LPC(16:1)	493.31684	1.00476	HMDB0010383	
⑩	0.78		129.1371	?	–	–	–	–	
⑪	8.05		468.3062	C22H46NO7P	LPC(14:0)	467.30119	1.00501	HMDB0010379	
⑫	7.66		415.2041	?	–	–	–	–	
⑬	1.47		123.0529	C6H6N2O	Nicotinamide	122.04801	1.00489	HMDB0001406	
⑭	8.88		496.3358	C24H50NO7P	LPC(16:0)	495.33249	1.00331	[HMDB0240262]	
⑮	8.71		292.6478	?	–	–	–	–	

a Monoisotopic mass of the most abundant isotopic species.

b Accurate mass (measured) – exact mass (calculated).

c Human Metabolome DataBase; ?, not yet assigned; –, not applicable; [HMDB0240262] Computerized identification by protonated exact mass within ±5 ppm mass error showed that LPC(16:0) was separated into two peaks with retention times of 8.88 (⑭) and 9.11 min (②). Therefore, we further validated the identity of candidate metabolites using authentic 1-palmitoyl-sn-glycero-3-phosphocholine. As a result, we were able to confirm the identity of ② as 1-palmitoyl-sn-glycero-3-phosphocholine. Hence, metabolite ⑭ may be 2-palmitoyl-sn-glycero-3-phosphocholine but could not be identified yet.

The fatty acids esterified in LPLs, which contribute relatively more to the GGA effect, were mostly polyunsaturated such as 20:3 and 20:4, and hydroxylated 20:3 was also observed (Table 1). Saturated (16:0, 14:0) and mono-unsaturated (16:1) fatty acids were found in LPLs, which contribute relatively less to the GGA effect, except that LPC(16:0) was an exceptional biomarker with a high contribution.

### 3.3 Kinetics of LPL up-regulation induced by GGA treatment

*S-plot analysis:* On the basis of the combination of retention time in UPLC and the observed accurate mass, the LPLs presented in Table 1 were traced on time-course S plots (Supplementary Fig. S1). LPC(20:4) (④) and LPE(20:4) (⑤) were detected in the upper-right region of the first quadrant of the S-plot already at 2 h after GGA treatment and were continuously detected at almost the same position at 4 and 8 h. However, LPC(16:0) (②) and LPC(16:1) (⑨) appeared in the upper-right corner of the S plot only after 2 h, and LPC(20:3) (①) and LPC(OH20:3) (③) appeared in the upper-right part of the S plot only after 8 h.

*Semi-quantitative analysis:* A calibration curve for LPC(16:0) was prepared by UPLC-Q-Tof/MS using 1-palmitoyl-*sn*-glycero-3-phosphocholine under the same conditions as in the metabolomics analysis. The mass signal intensity of the peak area increased linearly from 80 to 20,000 in the concentration range of LPC(16:0) from 0.3 to 40 pmoles (Supplementary Fig. S2). Therefore, we targeted eight LPLs in Table 1 for semi-quantitative analysis using their respective retention times and exact masses, assuming that the mass signal intensity was linear with the concentration of LPLs injected.

**Fig. 3:**
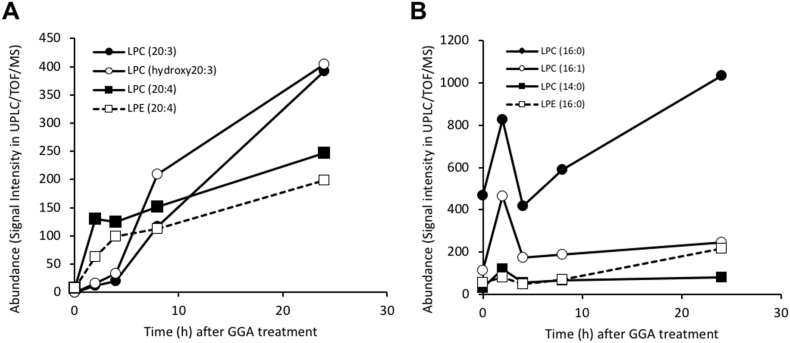
Time-dependent changes in the concentrations of various LPLs in HuH-7 cells after GGA treatment. **A**: Time-dependent changes in LPLs containing polyunsaturated fatty acids (20:3, 20:4). **B**: Time-dependent changes in LPLs containing saturated fatty acids (14:0, 16:0) or monounsaturated fatty acid (16:1).

As shown in Fig. 3A, LPC(20:4) (④) and LPE(20:4) (⑤) were barely detectable before GGA treatment, but were significantly increased at 2 h after GGA treatment, and then gradually and continuously increased up to 24 h. LPC(20:3) (①) and LPC(OH20:3) (③) were almost undetectable at 0 h, and showed only a slight increase up to 4 h, a sharp increase at 8 h, and a further increase above the two 20:4-containing LPL levels at 24 h.

Conversely, LPC(16:0) (②) was present in relatively large amounts even before GGA treatment and showed a transient 1.8-fold increase at 2 h, returning to the original value at 4 h and then gradually increasing to reach 2.2-fold of the basal level at 24 h (Fig. 3B). Similarly, LPC(16:1) (⑨) showed a transient increase of 4.1-fold at 2 h, and then gradually increased to 2.1-fold at 24 h. LPE(16:0) (⑦) and LPC(14:0) (⑪) increased 2.5- and 4-fold relative to the basal levels at 24 h, respectively.

## 4 Discussion

In this paper, we undertook a metabolomics approach to observe the changes in intracellular metabolites of HuH-7 cells after 24-h GGA treatment. To our surprise, out of the top 11 identified biomarkers found to specifically increase in GGA-treated cells, nine were all LPLs. Although this was a completely unexpected result, a semi-quantitative analysis of the temporal variation of the eight LPLs after GGA treatment showed that LPC and LPE containing polyunsaturated or saturated fatty acids were already increased at 2 h and then further increased up to 24 h.

**Fig. 4:**
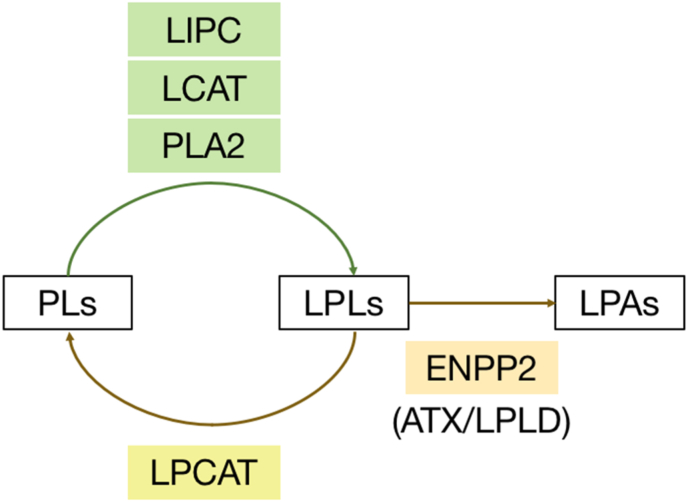
Enzymes involved in the increase and decrease in intracellular lysophospholipids in human hepatocytes. PLs, phospholipids; LPLs, lysophospholipids; LPAs, lysophosphatidic acids; LIPC, lipase C; LCAT, lecithin cholesterol acyltransferase; PLA2, phospholipase A2; LPCAT, lysophosphatidylcholine acyltransferase; ENPP2, ectonucleotide pyrophosphatase/phosphodiesterase 2; ATX, autotaxin; LPLD, lysophospholipase D.

In general, the increase in intracellular LPLs could be due to either an increase in LPLs production, a decrease in their degradation, or both (Fig. 4) [15,16]. Here, we first considered the possibility of increased synthesis of LPLs: Because GGA-induced cell death is a type of inflammatory cell death with the cytosolic calcium surges [11,12] and nuclear translocation of cytosolic NF-κB [12], activation of phospholipase A2 (PLA2), which is well known to be activated by calcium [17] and transactivated by NF-κB [18], was expected. However, because PLA2 is an enzyme known to release arachidonic acid in the *sn*-2 position and contributes to the production of eicosanoids [19], it is highly unlikely that the activated PLA2 enhances the production of LPC(20:4) and LPE(20:4). The same is true for lecithin cholesterol acyltransferase (LCAT), which transfers the fatty acid in the *sn*-2 position of PC to cholesterol [20]. Lipase C (LIPC) is a hydrolytic enzyme that liberates fatty acids in the *sn*-1 position [21]. When LIPC is activated, LPLs containing the most abundant 18:2 and 18:1 should increase. UPLC/Q-Tof/MS tracing using the exact mass of each LPL on the protonated assumption detected some LPCs and LPEs containing C18-series fatty acids, but all of them except LPC(18:1) showed a gradual, but not dramatic, increase after GGA addition (Supplementary Fig. S3). From these perspectives, GGA-induced up-regulation of the LPLs cannot be explained by the activation of any of the three enzymes, PLA2, LCAT, and LIPC.

Next, we considered the suppression of the decreasing system of LPLs. There are two possible mechanisms: the suppression of LPC acyltransferase (LPCAT) and the suppression of ectonucleotide pyrophosphatase/phosphodiesterase 2 (ENPP2) (see Fig. 4). It has been reported that the expression of either LPCAT1 or ENPP2 is increased and some LPCs are decreased in diverse human tumors including hepatoma [22], melanoma [23], and acute myeloid leukemia [24]. LPCAT catalyzes the reacylation of LPLs at the *sn*-2 position. LPCAT3, which is abundant in the liver, prefers 18:2- or 20:4-acyl CoA as a donor of fatty acids to LPC and LPE [15]. Therefore, LPLs, which are increased by inhibition of LPCAT, would contain mostly saturated fatty acids. Conversely, ENPP2 acts on any LPL such as LPC and LPE, to produce LPA [16]. Therefore, when ENPP2 is suppressed, LPC and LPE containing saturated or unsaturated fatty acids would increase.

On the basis of the above considerations, it is reasonable to speculate that the GGA-induced increase in multiple LPLs could be mainly due to the down-regulation of ENPP2. In other words, if ENPP2 was suppressed by GGA, it could well explain GGA-induced increase in intracellular LPLs such as LPC(16:0), LPC(20:4), LPE(16:0), and LPE(20:4). Of note, ENPP2 expression has recently been considered to be very important in hepatoma development [25], including replication of hepatitis B virus [26] and hepatitis C virus [27]. Moreover, high ENPP2 expression in hepatoma is detected in patients with histological grade II/III [28]. Therefore, ENPP2 is expected to be a promising target for hepatoma suppression, and the possibility of reducing ENPP2 activity by GGA will pave the way for future hepatoma prevention.

Finally, we discuss the role of the GGA-induced increases in the LPLs identified in the present study in GGA-induced pyroptosis. We reported that GGA-induced cell death is very similar to the well-known lipotoxicity caused by saturated fatty acids [11]. The GGA-induced UPR^ER^ is inhibited by oleate co-treatment, which is similar to the UPR^ER^ induced by saturated fatty acids [11]. Of note, Kakisaka et al. reported that palmitate-mediated lipotoxicity in HuH-7 cells is associated with UPR^ER^, which is caused by LPC(16:0) [29]. In fact, they showed that palmitate is not required to induce UPR^ER^ and lipotoxicity in HuH-7 cells if only LPC(16:0) is added to the medium [29]. Interestingly, the unbiased metabolomics analysis in the present study showed that LPC(16:0) was transiently increased 2 h after the addition of GGA to HuH-7 cells. Hence, it can be explained that intracellular LPC(16:0) increased by GGA may induce UPR^ER^, resulting in eventual cell death. In addition, a previous report showing that LPCs cause pro-inflammatory changes by activating TLR4 [30] and a recent report demonstrating that LPC induces pyroptosis [31] both strongly support the idea that the increase in a group of LPLs by GGA forms part of the mechanism for pyroptotic cell death via TLR4 signaling through UPR^ER^.

In conclusion, we performed an unbiased metabolomics analysis in GGA-treated cells, which revealed GGA-induced up-regulation of several LPLs including LPC(20:4), LPE(20:4), LPC(16:0), and LPE(16:0). The possible mechanism by which GGA increases these LPLs and the involvement of LPLs in GGA-induced pyroptosis were discussed. LPLs are a class of lipids that are not only localized inside cells but also secreted outside cells. In particular, the majority of plasma LPLs are thought to be secreted by the liver [32]. Therefore, analyzing the profile and concentration of LPLs in the blood during clinical hepatoma treatment may allow monitoring of cell death in hepatoma cells.

## Data availability

Data will be made available on request.

## Author statemenr

Chieko Iwao: Methodology, Formal analysis, Investigation, Writing - Methods & Results Sections, Visualization.Yoshihiro Shidoji: Conceptualization, Methodology, Writing - Introduction & Discussion Sections, Review & Editing, Visualization, Supervision, Project administration, Funding acquisition.

## Declaration of competing interest

The authors declare that they have no known competing financial interests or personal relationships that could have appeared to influence the work reported in this paper.
