# Supplementary material for: A rapid increase in lysophospholipids after geranylgeranoic acid treatment in human hepatoma-derived HuH-7 cells revealed by metabolomics analysis
Source: Biochem Biophys Rep. 2021 Nov 24;28:101176. doi: 10.1016/j.bbrep.2021.101176 (PMC8626837; doi:10.1016/j.bbrep.2021.101176)
Supplement: Multimedia component 1 [file mmc1.docx]

(Supplement)

A rapid increase in lysophospholipids after geranylgeranoic acid treatment in human hepatoma-derived HuH-7 cells revealed by metabolomics analysis

Yoshihiro Shidoji and Chieko Iwao

Molecular and Cellular Biology, Graduate School of Human Health Science, University of Nagasaki, Nagayo, Nagasaki 851-2195, Japan

Corresponding author: Yoshihiro Shidoji, [shidoji@sun.ac.jp](mailto:shidoji@sun.ac.jp)

**Supplementary Figures**


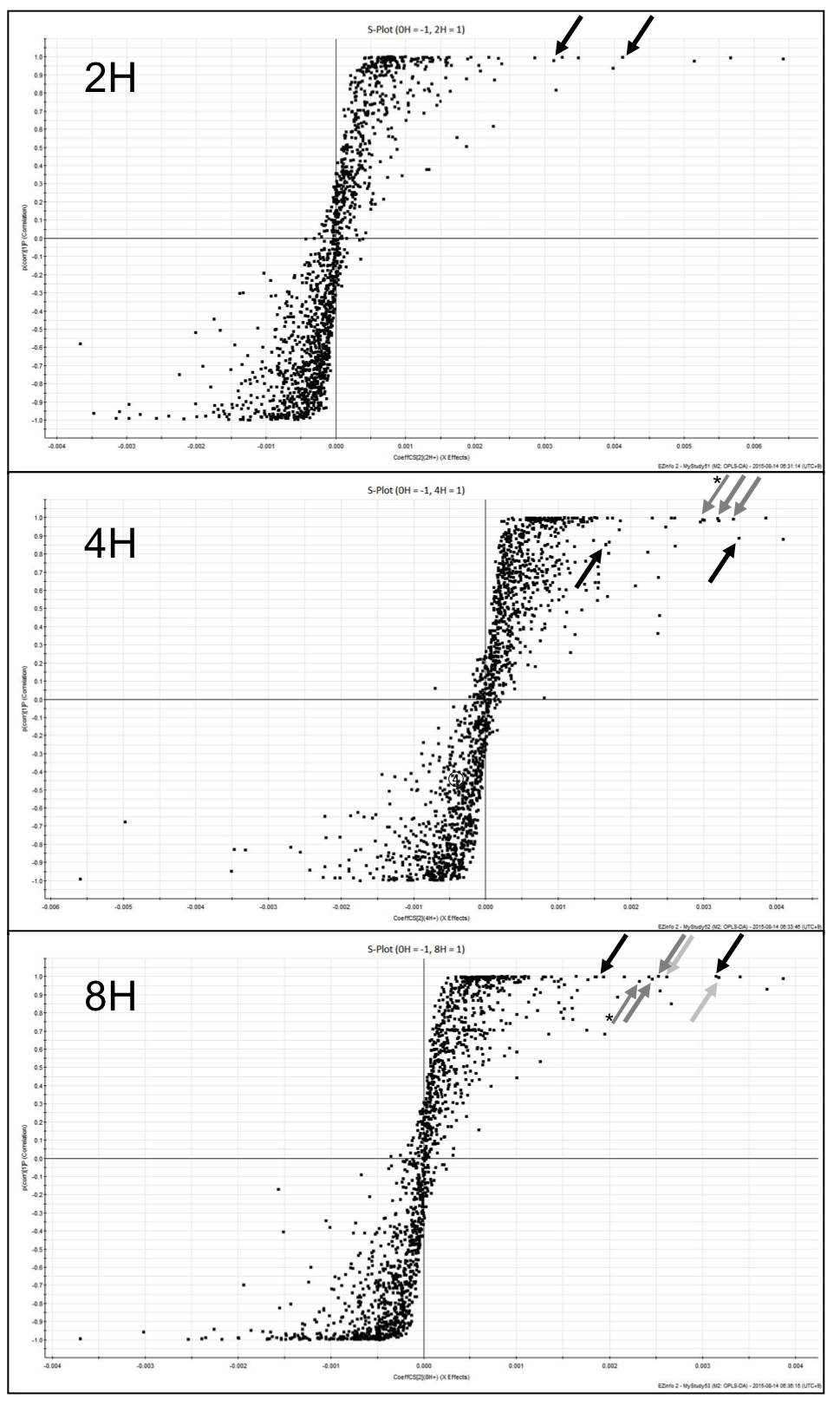


④

⑤

④

⑤

④

③

①

➁

⑨

⑤

**Supplementary Figure S1:** OPLS-DA S-plots: 2-h GGA treated cell extracts vs 0-h control (2H), 4-h GGA treated vs 0-h control (4H), and 8-h GGA treated vs 0-h control (8H). The circled numbers indicate the same circled numbers shown in Figure 2B and Table 1.

**Supplementary Figure S2:** Calibration curve for 1-palmitoyl-*sn*-3-phosphocholine (LPC (16:0)) by UPLC-Q-Tof/MS using exact mass.

**Supplementary Figure S3:** Time-dependent changes in LPLs containing C18-series fatty acids (18:0, 18:1, 18:2, and 18:3) after GGA treatment.
