# Supplementary figures and images for: A rapid increase in lysophospholipids after geranylgeranoic acid treatment in human hepatoma-derived HuH-7 cells revealed by metabolomics analysis
Source: Biochem Biophys Rep. 2021 Nov 24;28:101176. doi: 10.1016/j.bbrep.2021.101176 (PMC8626837; doi:10.1016/j.bbrep.2021.101176)

## Slide 1
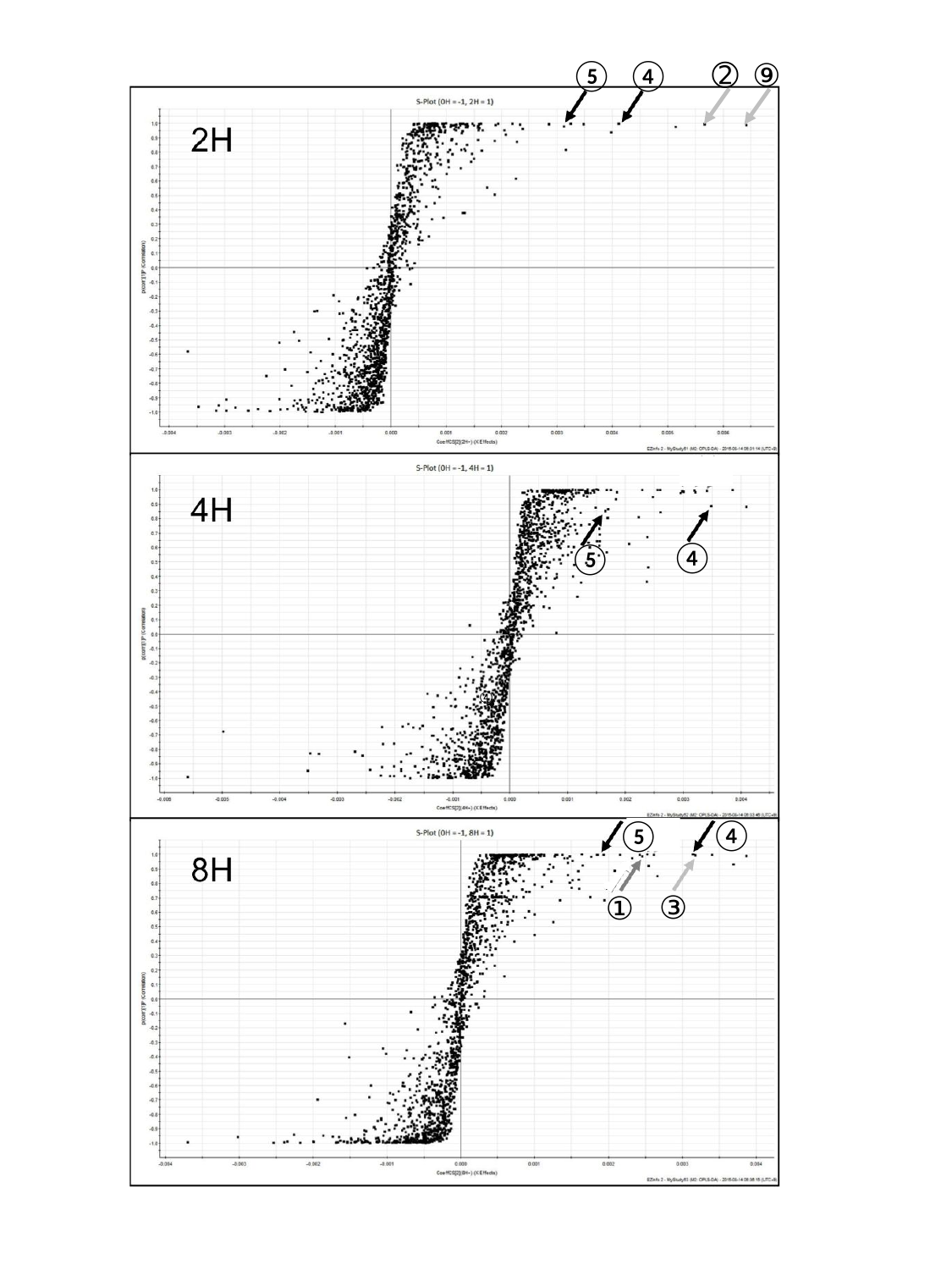

⑤
④
➁
⑨
④
⑤
④
⑤
③
①

Supplement: Multimedia component 2 [file mmc2.pptx]

## Slide 1
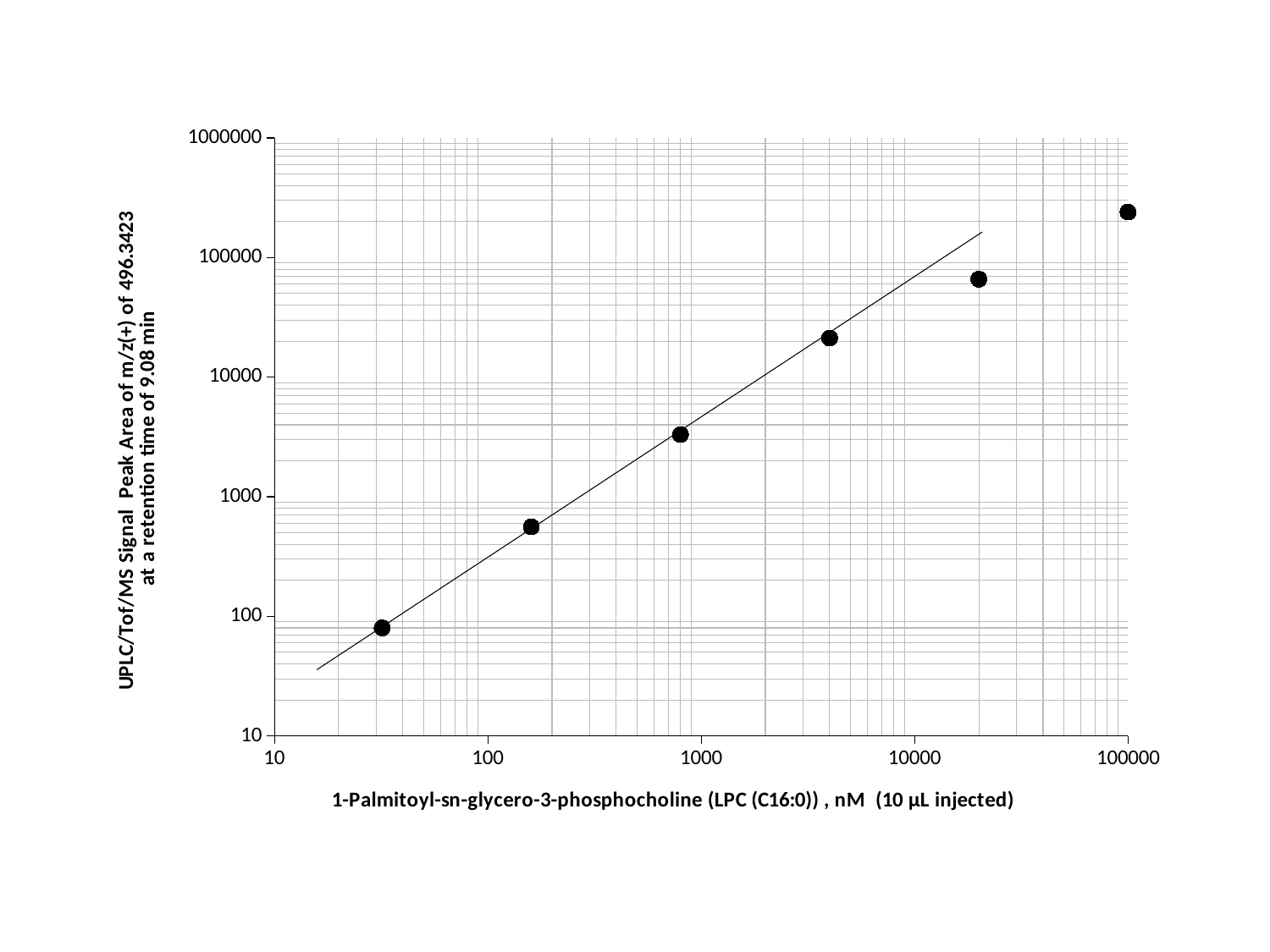

### Chart
| Category | TOF-MS Area |
|---|---|

Supplement: Multimedia component 3 [file mmc3.pptx]

## Slide 1
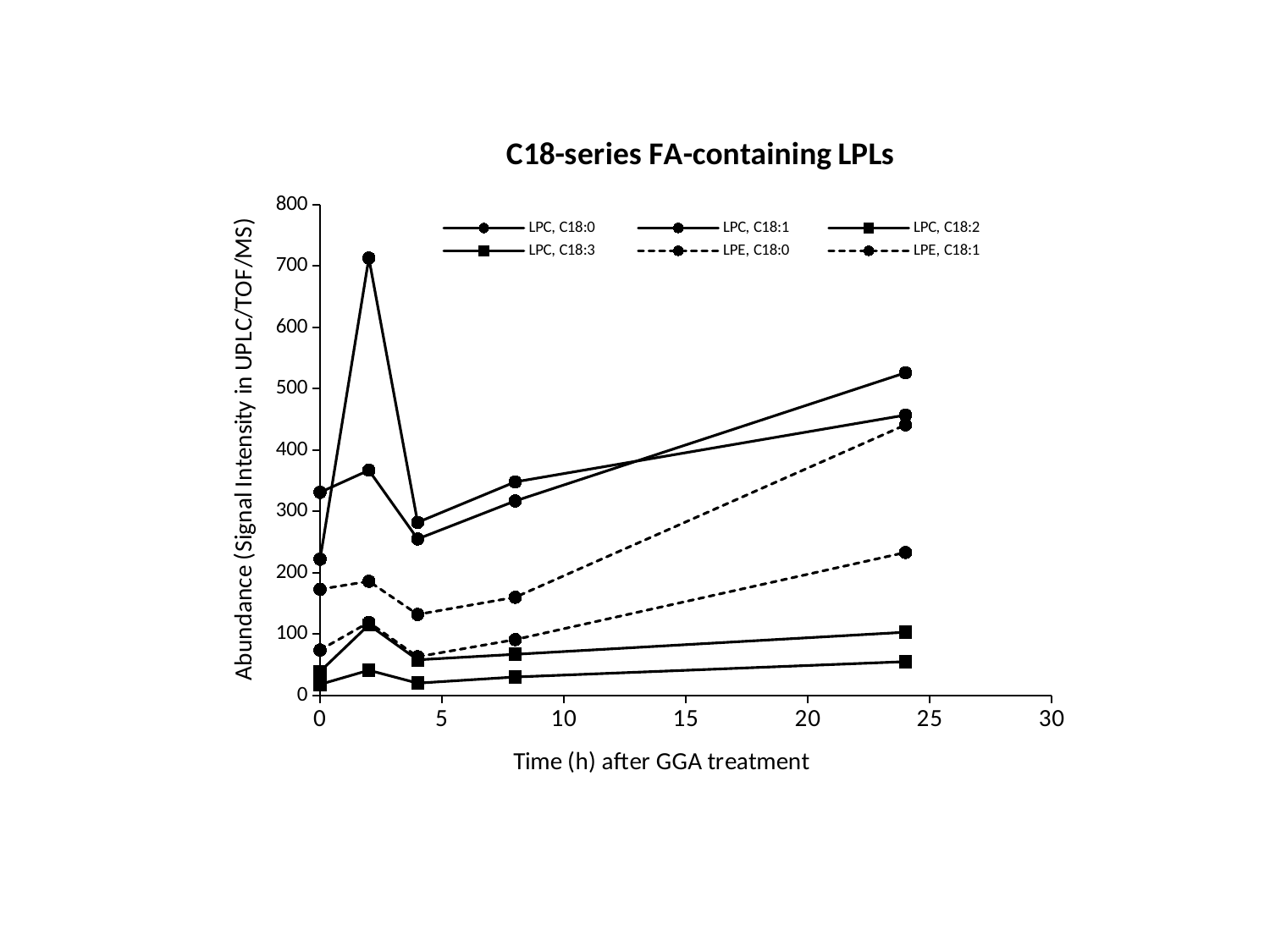

### Chart: C18-series FA-containing LPLs
| Category | LPC, C18:0 | LPC, C18:1 | LPC, C18:2 | LPC, C18:3 | LPE, C18:0 | LPE, C18:1 |
|---|---|---|---|---|---|---|

Supplement: Multimedia component 4 [file mmc4.pptx]
